# *Nosema* spp. infection and its negative effects on honey bees (*Apis mellifera iberiensis*) at the colony level

**DOI:** 10.1186/1297-9716-44-25

**Published:** 2013-04-10

**Authors:** Cristina Botías, Raquel Martín-Hernández, Laura Barrios, Aránzazu Meana, Mariano Higes

**Affiliations:** 1 Laboratorio de Patología Apícola, Centro Apícola Regional, CAR, Junta de Comunidades de Castilla La Mancha, Marchamalo 19180, Spain; 2 Instituto de Recursos Humanos para la Ciencia y Tecnología, INCRECYT, Parque Científico de Albacete, Albacete, Spain; 3 Departamento de Estadística, CTI, Consejo Superior Investigaciones Científicas, Madrid, 28006, Spain; 4 Departamento de Sanidad Animal, Facultad de Veterinaria, Universidad Complutense de Madrid, Madrid, 28040, Spain

## Abstract

Nosemosis caused by the microsporidia *Nosema apis* and *Nosema ceranae* are among the most common pathologies affecting adult honey bees. *N. apis* infection has been associated with a reduced lifespan of infected bees and increased winter mortality, and its negative impact on colony strength and productivity has been described in several studies. By contrast, when the effects of nosemosis type C, caused by *N. ceranae* infection, have been analysed at the colony level, these studies have largely focused on collapse as a response to infection without addressing the potential sub-clinical effects on colony strength and productivity. Given the spread and prevalence of *N. ceranae* worldwide, we set out here to characterize the sub-clinical and clinical signs of *N. ceranae* infection on colony strength and productivity. We evaluated the evolution of 50 honey bee colonies naturally infected by *Nosema* (mainly *N. ceranae*) over a one year period. Under our experimental conditions, *N. ceranae* infection was highly pathogenic for honey bee colonies, producing significant reductions in colony size, brood rearing and honey production. These deleterious effects at the colony level may affect beekeeping profitability and have serious consequences on pollination. Further research is necessary to identify possible treatments or beekeeping techniques that will limit the rapid spread of this dangerous emerging disease.

## Introduction

Honey bees are key generalist pollinators that live in large perennial colonies, ensuring high levels of local pollination throughout the flowering season. *A. mellifera* considerably contributes to general crop pollination, the benefits of which are substantially higher than those resulting from honey production [1]. However, it has been reported that, on a global level, honey bees are primarily reared to produce honey and other marketable products and thus, the economics of honey production influence the global dynamics of bee management more than their influence on agricultural and biological pollination [2,3]. Honey is by far the most common and best known product from honey bee colonies, both from the quantitative and economic point of view [4,5], and it represents an essential commodity worldwide which adds nutritional variety to human diets [3]. Honey production and trade is a source of finance with global production reaching 1.07 million tonnes in 2007 [6]. In Spain, which has the largest number of bee colonies, the highest honey production and the second largest population of professional beekeepers after Greece from the European Union [7], honey production is estimated at 31 800 thousand tonnes. Based on the average price of honey in Spain in 2007 (€ 2377 per tonne) [8], this activity is valued at approximately € 75.7 million. Given these figures, it is clear that any loss in honey production is an important concern for professional beekeepers, particularly in the most productive countries.

Several factors affect honey production in the colony, including weather conditions [9], the availability of adequate foraging resources [10] and colony strength (i.e. brood production, size of the adult bee population, worker bee life expectancy and the individual productivity of workers) [11].

Honey bee parasites and pathogens have also been reported to negatively influence colony productivity [12,13], and some of the most damaging diseases of adult honey bees are varroosis caused by the mite *Varroa destructor*[14,15], and microsporidiosis caused by the spore-forming microsporidia *Nosema apis* and *Nosema ceranae*[16-18]. Both these microsporidian species infect the midgut (ventriculus) of the honey bees’ digestive tract, although they cause two different diseases, with distinct epidemiological, clinical and pathological characteristics [5,19]. Since the relatively recent discovery of *N. ceranae* infection in *A. mellifera*[20], the number of studies analysing the impact of the infection by this microsporidium (namely Nosemosis type C) on honey bees at the individual level has increased markedly [21-24]. The effect of nosemosis type C at the colony level has also been evaluated [17,25,26], primarily focussing on the possible colony collapse in response to infection, and disregarding the potential sub-clinical effects on parameters such as colony strength and productivity. Since several studies have described negative impacts of *N. apis* infection on these colony parameters [27-29], and in the light of the high prevalence of *N. ceranae* worldwide [19,30], we sought to identify the clinical and sub-clinical signs of *Nosema* infection on colony strength and productivity when *N. ceranae* is the dominant species, which may affect beekeeping profitability and the efficiency of honey bee pollination.

## Materials and methods

### Honey bee colonies

In this study, 50 homogeneous colonies of *Apis mellifera iberiensis* in the experimental apiaries located at the “Centro Apícola de Marchamalo” (CAR) were monitored monthly from September 2007 to December 2008. Thereafter, the presence/absence of the queen, colony mortality and clinical and sub-clinical sings of any pathology were recorded each month until December 2010. All colonies were acquired as nuclei (5 combs with worker bees and a queen) from the same professional beekeeper in April 2007, and they were left to develop and adapt to the field conditions of the study until June 2007, when they were introduced into the new hives. At the start of the study, neither colony showed clinical signs of chalkbrood, American or European foulbrood. Furthermore, the absence of *V. destructor* was recognised after examining the bees according to OIE methods [31] and *Nosema* spp. was not detected in the colonies when bee samples (*n* ≥ 30 foragers) were analysed using a specific duplex PCR [32].

The areas surrounding the hives contained no genetically modified agricultural crops, and no insecticides such as fipronil or imidacloprid were in use.

The colonies were placed close to another apiary containing colonies naturally parasitised by *N. ceranae* and/or *N. apis*, which acted as a natural source of infection.

*V. destructor* parasitisation was controlled twice a year (Apivar^®^ a.m. amitraz) to avoid the negative effects of this parasite on colony health. This experimental research follow the guidelines of the European Medicines Agency and the methods have been approved by the Spanish National Institute for Agricultural and Food Research and Technology (INIA).

### Field assay procedure

Upon detection of *Nosema* spp. in September 2007 in all 50 colonies (100% *N. ceranae*-positive; 50% co-infected with *N. apis*, see Additional file 1), the colonies were randomly assigned to 5 experimental groups, each containing 10 colonies distributed in two different apiaries to reduce the risk of reinfection with *Nosema* spp. of the treated colonies through the contact with the untreated ones. Both apiaries were situated 500 m away from one another and at a similar altitude (Apiary 1: 722 meters; Apiary 2: 720 meters), being surrounded by the same type of flora (degradation stage of holm oak-Aleppo pine forest surrounded by cereal crops; see Additional file 2). In Apiary 1 (40°40^′^45.18^′^N, 3°13^′^1.01″W), 3 experimental groups were treated for infection by *Nosema* spp. (Fumidil-B^®^; 4 doses of 30 mg fumagillin per colony administered at one week intervals: [33]). Accordingly and as summarized in Table 1, colonies of group 1 T received one treatment (Autumn 2007), those of group 2 T were treated twice (Autumn 2007/Spring 2008), while group 4 T received one treatment per season (Autumn 2007/Winter 2008/Spring 2008/Summer 2008). In Apiary 2 (untreated colonies; 40°40^′^59.25″N, 3°12^′^54.65″W), two experimental groups were established: colonies of group CS received one application of sugar syrup (vehicle; 1:1 distilled water-sugar) each season (Autumn 2007/Winter 2008/Spring 2008/Summer 2008), while group C (control group) was made up of 10 unmanaged colonies. Due to the random separation of the 50 colonies in 5 groups, the co-infected colonies (50% of the total) were not equally distributed within the groups (see Additional file 1), but all groups contained between 3 and 6 co-infected colonies.

**Table 1:** Chronology of interventions in all colonies (FB = Forager Bees; HB = House Bees; ALL = All groups)

**INTERVENTIONS IN THE COLONIES**	**Sep 07**	**Oct 07**	**Nov 07**	**Jan 08**	**Feb 08**	**Apr 08**	**May 08**	**Jun 08**	**Jul 08**	**Aug 08**	**Sept 08**	**Oct 08**	**Nov 08**	**Dec 08**
Fumagillin application		1 T 2 T 4 T		4 T			2 T 4 T			4 T				
Syrup application		CS		CS			CS			CS				
FB collection and PCR analysis of composite samples	ALL	ALL	ALL	ALL	ALL		ALL		ALL	ALL	ALL			ALL
FB collection and PCR analysis of individual bee samples		ALL	ALL				ALL		ALL					
HB collection and PCR analysis of composite samples		ALL	ALL				ALL		ALL					
HB collection and PCR analysis of individual bee samples		ALL	ALL				ALL		ALL					
Data collection of adult bee population and brood rearing		ALL	ALL	ALL	ALL	ALL	ALL	ALL	ALL	ALL		ALL	ALL	ALL
Data collection of honey production											ALL			

The experimental groups of colonies were established as follows: group 1 T (treatment in autumn 2007), group 2 T (treatment in autumn 2007 and spring 2008), group 4 T (treatment in autumn 2007, winter, spring and summer 2008), group CS (sugar syrup in autumn 2007, winter, spring and summer 2008) and group C (unmanaged colonies).

Both sugar-syrup and fumagillin were administered in plastic bags placed over the brood chamber and their consumption was assessed weekly [33]. Fumagillin consumption was calculated on the basis of 30 mg fumagillin per 250 mL syrup ingested. On the day a new dose was administered, the remaining unconsumed doses were removed from the colony and weighed.

From September 2007 until December 2010, clinical signs indicative of pathologies other than *Nosema* disease, the presence of a queen and colony mortality were recorded on each visit to the apiaries (Table 1).

### Monitoring of *Nosema* spp. infection in the experimental colonies

#### Detection of infection (composite samples)

To evaluate *Nosema* spp. infection in the colonies, samples of forager bees (*n* ≥ 30 bees per sample) and house bees (*n* ≥ 30 bees) were collected at noon, as described previously [34]. Forager bee samples were collected 10 times, approximately once a month, between September 2007 and December 2008 (Table 1). House bee samples were only collected before and after the interventions in autumn 2007 and spring 2008. DNA was extracted from the composite samples collected and PCR was performed as described previously [35].

#### Percentage of infected bees before and after interventions

The percentage of worker bees infected by *Nosema* spp. was also evaluated in each colony before and after the interventions in the autumn 2007 and spring 2008 in order to determine the effect of the different interventions on the evolution of *Nosema* spp. infection at the beginning of the assay (autumn) and during the period of most activity in the colonies (spring) [36]. Samples of forager bees (*n* ≥ 20) and house bees (*n* ≥ 20) were collected from each colony as described above, one week before the first dose of treatment and two weeks after the last dose (Table 1).

For forager bee samples, the percentage of infected bees was determined by individual PCR analysis of the 20 forager bees from each of the studied colonies, following the procedures described in a previous assay [35].

For house bees, the percentage of infected bees was determined in randomly selected colonies that remained infected after the interventions, using the same laboratory methods described above for forager bees.

### Colony strength

The adult bee population and brood production (i.e. uncapped and capped brood) were evaluated throughout the assay (from October 2007 to December 2008, Table 1) by analyzing the frames covered by bees and quantifying the number of brood cells [17,37]. Both the adult bee population and the number of brood cells were evaluated each month (except for the months of December 2007, and March and September 2008), for a total of 12 measurements.

The proportion of brood cells (NC) to the number of combs covered by bees (P) was also calculated to determine the ratio of brood to bees (NC/P).

### Honey production

The honey production of each colony was evaluated at the harvesting season (September 2008) by separately weighing all the frames with honey from each colony and calculating the total amount of honey stored in them from the difference in comb weight before and after honey extraction [36]. Only those colonies alive at the harvesting season were used for the analysis.

### Statistical analysis

Dependent variables studied were the following: percentage of infected bees, adult honey bee population, number of brood cells, proportion of brood cells per bee combs and honey production. They were normalized with linear transformations. Two factors were taken into account, one fixed factor (group) and the time effect. We ran Generalized Estimation Equations with probability distribution normal, link function identity, subject effect colony and within-subject effect time point.

Since the presence of interaction in the model revealed that the effects of interventions varied with time effect, the mean values of the different parameters (percentage of infected bees, adult honey bee population, number of brood cells and proportion of brood cells per bee combs) were compared per group and time point using a one-way analysis of variance (ANOVA). To evaluate the effect of the interventions in honey production, the mean values recorded from each group at the harvesting season (Sept. 08) were also compared using one-way ANOVA. Where necessary, ANOVA was followed by Bonferroni or Tamhane *post hoc* tests, depending on the homogeneity of variance in each case (determined using Levene’s test).

To assess the effect of the interventions in the colonies we ran a paired *t* test comparing the percentage of infected bees before and after interventions both in autumn 07 and in spring 08.

A Student’s *t* test was used to compare the prevalence of *N. apis* versus *N. ceranae* in individual forager bee samples.

In all statistical studies, differences were considered significant when α ≤ 0.05.

The Spearman’s rank correlation test was used to determine the degree of dependence between the percentage of *N. ceranae* infected bees and: (a) population size; (b) the amount of brood cells; (c) honey production in the corresponding hives; and (d) the amount of fumagillin consumed in the case of treated colonies (groups 1 T, 2 T and 4 T in autumn 2007, and groups 2 T and 4 T in spring 2008). The same analysis was performed with the percentage of *N. apis* infected bees. These correlations were performed as a function of the percentage of infected bees from November 2007 and July 2008. Correlations were considered significant when α ≤ 0.05 (2-tailed test).

All analyses were carried out using SPSS 18.0 software.

## Results

### Monitoring of *Nosema* spp. infection in experimental colonies

#### Detection of infection (composite samples)

##### Forager bees

**Figure 1:**
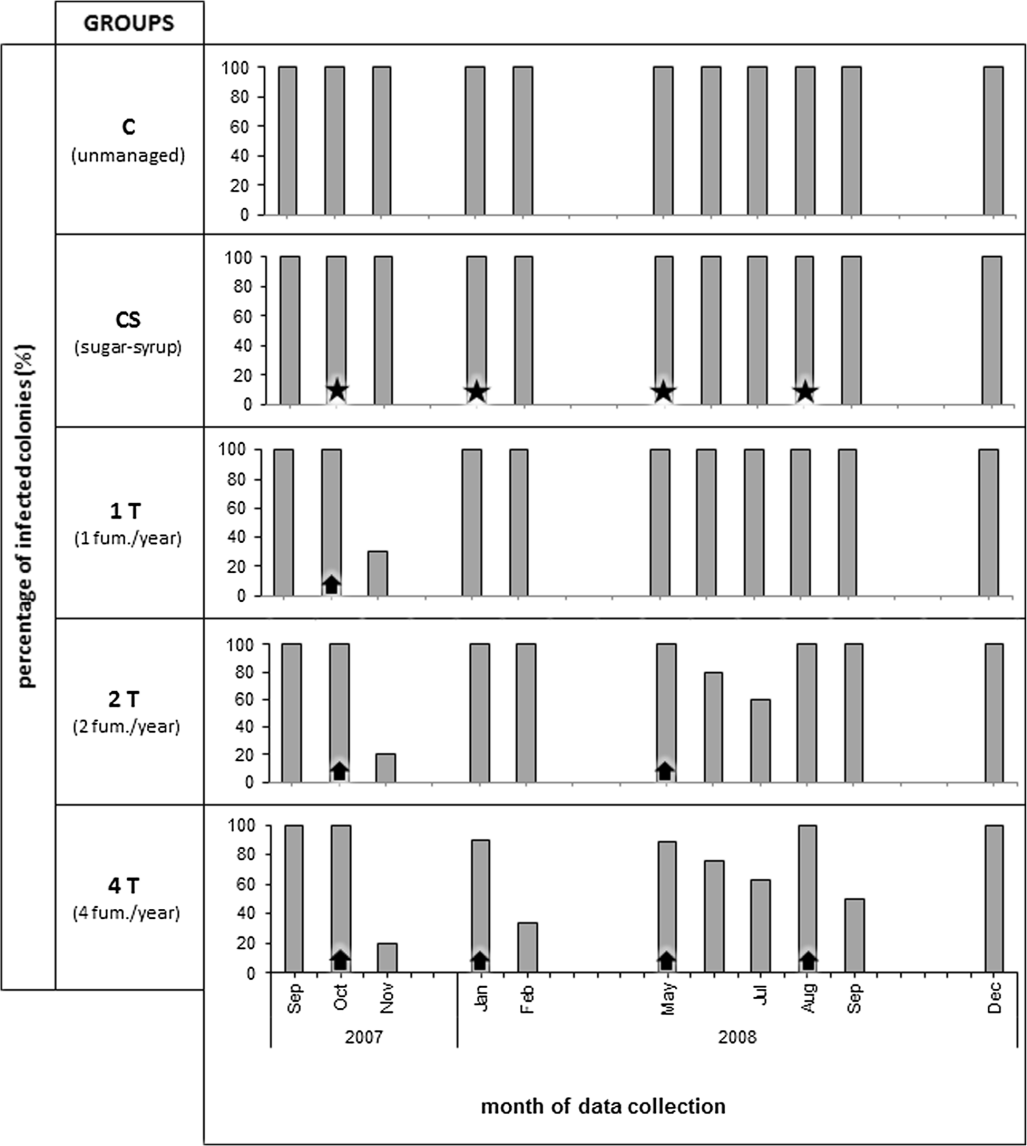
**Percentage of colonies infected by *Nosema *spp. during the assay, as a function of the analysis of composite forager bee samples (*n*** **≥ 30 bees per sample).** The time points at which fumagillin (arrows) and sugar-syrup (stars) were administered are indicated.

The 50 composite forager bee samples analysed all tested positive for *N. ceranae* infection at the beginning of the assay in September 2007, with 25 (50%) also exhibiting co-infection with *N. apis*. Analysis of the composite forager bee samples throughout the year (Sept. 2007 to Dec. 2008) primarily revealed infection by *N. ceranae* alone in the *Nosema*-positive colonies (314 out of 413 *Nosema*-positive samples; 76%), although co-infection with *N. apis* was also detected (97 out of 413 *Nosema*-positive samples; 23.5%). By contrast, infection by *N. apis* alone was only detected in 2 colonies (0.5%). Both microsporidia were detected throughout the year and while the presence of *N. ceranae* was constant, that of *N. apis* was more evident in the autumn and spring months (see Additional file 1). All of the untreated colonies remained infected throughout the assay, whereas *Nosema* was not detected in some of the treated colonies after intervention (Figure 1).

##### House bees

**Figure 2:**
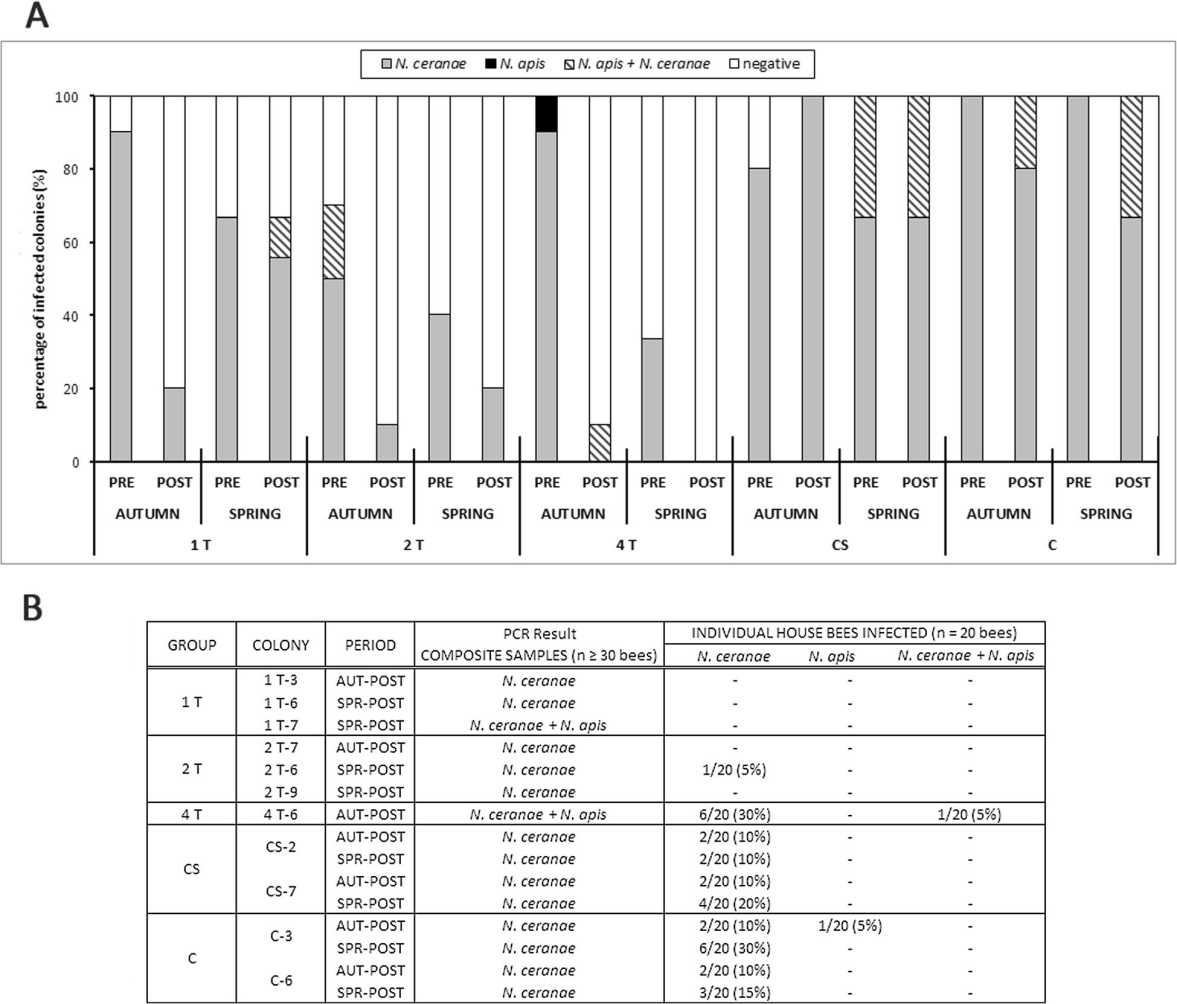
***Nosema *spp. infection of house bees. A**: Percentage of colonies infected by *Nosema* spp. in each group before and after the interventions in autumn 2007 and spring 2008 based on the PCR analysis of the composite samples of house bees. The experimental groups of colonies were established as follows: group 1 T (treatment in autumn 2007), group 2 T (treatment in autumn 2007 and spring 2008), group 4 T (treatment in autumn 2007, winter, spring and summer 2008), group CS (sugar syrup in autumn 2007, winter, spring and summer 2008) and group C (unmanaged colonies). **B**: Individual worker analysis (*n* = 20 house bees) in randomly selected colonies, and the corresponding results when composite samples from the same colonies were analysed (*n* ≥ 30 house bees). Hyphens indicate no PCR signal for this *Nosema* species in the individual bees analysed.

Before the interventions in October 2007, *N. ceranae* infection was evident in 43 of the colonies (86%), 2 of which (4%) were co-infected with *N. apis* (two colonies of group 2 T). No infection was detected in 5 colonies (10%) after analysing the composite house bee samples (1 colony of group 1 T, 3 colonies of group 2 T and 1 colony of group CS). The percentage of colonies infected by *Nosema* spp. was evaluated (Figure 2A) based on the analysis of composite house bee samples (*n* ≥ 30 bees) before and after the autumn 2007 and spring 2008 interventions.

#### Percentage of infected bees before and after intervention

##### Forager bees

**Figure 3:**
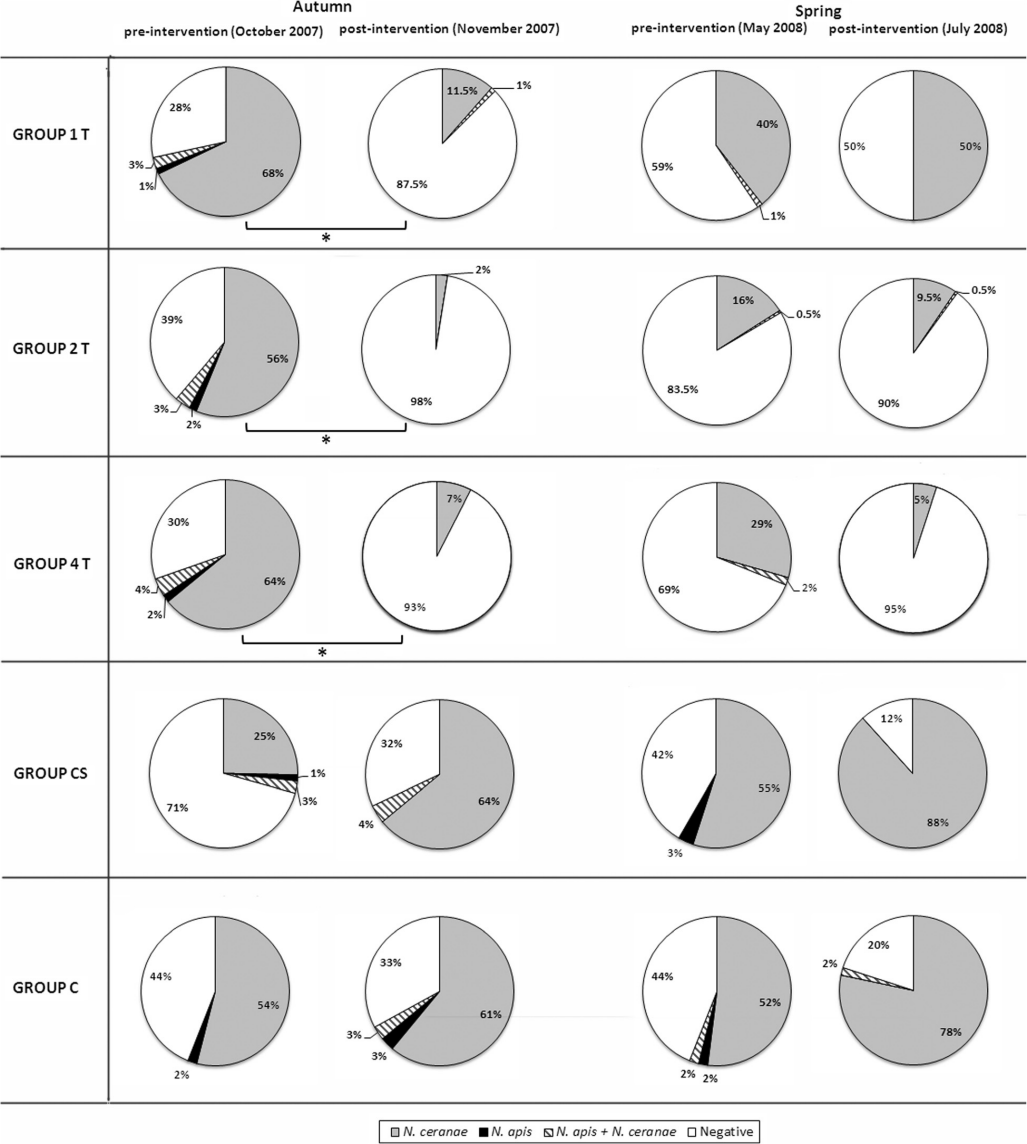
**Percentage of individual *Nosema-*infected forager bees per group (*n*** **= 200 bees individually analysed per group and time point; i.e. 20 bees from each of the 10 colonies of every group).** Analyses were performed in the autumn 2007 (pre- and post-intervention) and spring 2008 (pre- and post-intervention). Asterisk indicates significant differences found (*P* < 0.01) in the pre-post intervention interval.

The percentage of foragers infected with *Nosema* spp. before intervention (October 2007) differed significantly between groups (ANOVA, F = 4.39, *P* = 0.006; Figure 3, see Additional file 3), with fewer infected bees detected in the CS group with respect to groups 1 T and 4 T. However, after the treatment was applied in the corresponding groups (November 2007), groups 1 T, 2 T and 4 T exhibited significantly fewer infected forager bees (ANOVA, F = 21.1, *P* ≤ 0.001; Figure 3, Additional files 3 and 4) than in groups CS and C.

As for the effect of the treatment against *Nosema* in each group in autumn 2007, it significantly decreased the proportion of infected foragers in groups 1 T, 2 T and 4 T from October to November (paired *t* test, *P <* 0.001). Conversely, the rates of infection tended to increase from October to November in the untreated colonies (groups CS and C: Figure 3), although this effect was not statistically significant (paired *t* test, *P >* 0.05).

In May 2008, before the spring intervention in the corresponding colonies, group 2 T had a smaller proportion of infected bees (*P* < 0.05) than the control CS and C groups. Similarly, in early July, after treatment application in the corresponding groups (i.e. 2 T and 4 T) and syrup supply in group CS, the percentage of infected bees presented significant differences between groups (ANOVA, F = 46.3, *P* < 0.001), showing an infection rate significantly lower for groups 2 T and 4 T than that registered in control groups CS and C. In the 1 T group that received fumagillin in autumn 2007 but not in spring 2008, we detected a significantly lower proportion of infected bees than in the untreated groups CS (*P* = 0.001) and C (*P* = 0.01), although there were significantly more infected bees than in groups 2 T (*P* < 0.001) and 4 T (*P* < 0.001: Figure 3, see Additional file 3).

An intra-group evaluation of the percentage of bees infected in spring 2008 revealed a decreasing trend in the 2 T and 4 T groups and an increasing trend in the 1 T, CS and C groups between May and July (Figure 3, see Additional files 3 and 5). However, no significant differences were detected within any of the experimental groups (paired *t* test, *P* > 0.05) before or after treatment (2 T and 4 T) or syrup (CS) administration, or in the colonies that received no intervention (1 T and C).

Moreover, in the treated colonies, syrup with fumagillin consumption was inversely proportional to the percentage of *Nosema*-infected bees (autumn intervention, *r* = −0.54, *P* = 0.021; spring intervention, *r* = −0.69, *P* < 0.001; Spearman’s rank correlation). In addition, there was a significantly higher prevalence of *N. ceranae* over *N. apis* when the two *Nosema* species were analysed in individual forager bees (Figure 3), both in the autumn (F = 54.9, *P* < 0.001; Student’s *t-*test) and in the spring (F = 19.3, *P* < 0.001, Student’s *t-*test).

##### House bees

As indicated in the methods, only a small group of colonies that were infected after the composite sample analysis were randomly selected to evaluate the rate of infection. Less than 5% of bees were infected in all the colonies analysed from the treated groups after the intervention in October 2007 and May 2008, except for colony 4 T-6, in which 35% of the bees were infected with *N. ceranae* after the autumn treatment. In the untreated colonies the levels of infection were in the range of 10% to 30% after the autumn and spring interventions (Figure 2B).

##### Adult bee population size and brood production

**Figure 4:**
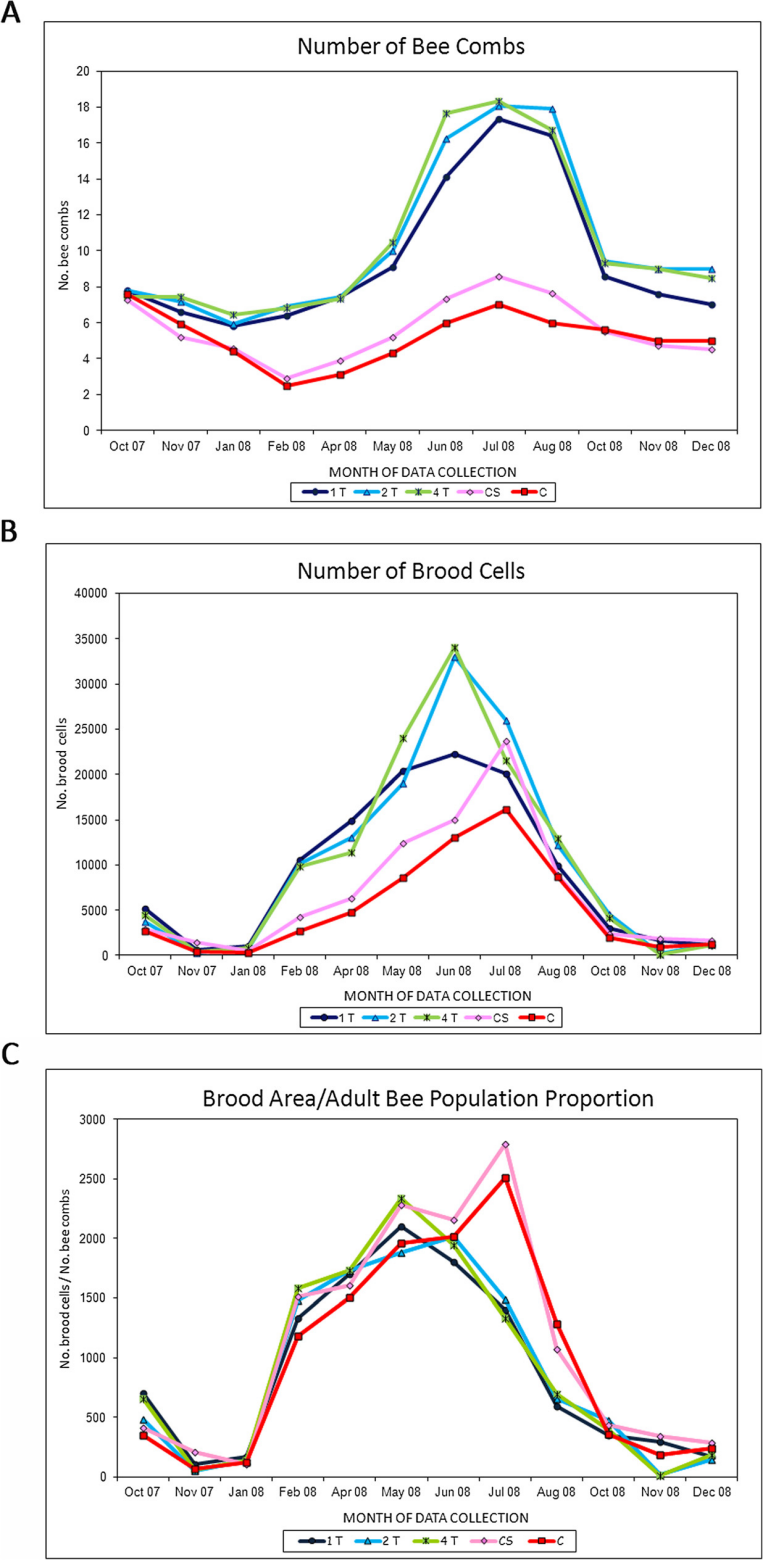
**Data collected from each colony group throughout the study. A**: Average number of bee combs per group per month. **B**: Average number of brood cells per group per month. **C**: Ratio of brood cells to bee combs in each group throughout the study.

At several stages during the study, the population size was larger in the colonies in which *Nosema* infection was controlled than in the untreated colonies. At the start of the study (October 2007) the adult bee populations were of equivalent sizes in all groups (ANOVA, F = 0.3, *P* = 0.8). However, a more pronounced and sustained drop in population size was observed in groups CS and C from the beginning of the study until February 2008, when the treated populations 1 T, 2 T and 4 T were significantly larger (ANOVA, F = 17.8, *P* < 0.001) than the control ones (CS and C: Figure 4A, see Additional file 6). Throughout the spring and summer months (from April to August), significantly larger populations were found in the treated 2 T and 4 T groups than in the control groups (CS and C, *P* < 0.001). Indeed, even the colonies in group 1 T (only treated in autumn 2007) were significantly more populated than those of the C group in July (*P* = 0.04) and August (*P* = 0.02). The maximum population was recorded at the same time in all the groups (July 2008: Figure 4A, see Additional file 6).

A significant negative correlation was detected between population size and the percentage of *N. ceranae* infected bees of any colony in July 2008 (*r* = − 0.57, *P* = 0.001; Spearman’s rank correlation), while such correlation was not observed in November 2007 (*r* = − 0.32, *P* = 0.07; Spearman’s rank correlation). No correlation was found between the percentage of *N. apis* infected bees and the population size in November 2007 (*r* = − 0.26; *P* = 0.12) or July 2008 (*r* = − 0.14; *P* = 0.23; Spearman’s rank correlation).

Like the adult bee population, the brood area was larger in the treated groups at several time points during the assay. Initially (October 2007), brood rearing was similar between groups (ANOVA, F = 0.6, *P* = 0.3), although significant differences appeared in February (ANOVA, F = 4.6, *P* = 0.003) and April 2008 (ANOVA, F = 5.4, *P* = 0.001) that were associated with a significant increase in brood cells in the 2 T and 4 T groups as compared with control CS and C groups (Figure 4B, Additional file 6). In May 2008, group C produced significantly less brood than groups 2 T (*P* = 0.03) and 4 T (*P* = 0.03), although one month later (June 2008) brood production was only greater in group 2 T with respect to the control groups CS (*P* = 0.002) and C (*P* = 0.004). At the end of the trial, in October 2008, group 2 T again contained significantly more brood cells than the control CS (*P* = 0.01) and C groups (*P* = 0.007). The brood production in the treated colonies (1 T, 2 T and 4 T) peaked in June, while that of the control colonies CS and C reached its highest level in July (Figure 4B, Additional file 6).

No significant correlation was observed between the proportion of bees parasitised by *N. ceranae* and the amount of brood cells in November 2007 (*r* = 0.28, *P* = 0.11; Spearman’s rank correlation) or July 2008 (*r* = − 0.19, *P* = 0.27; Spearman’s rank correlation), as resulted with the proportion of *N. apis* parasitised bees and the brood in November 2007 (*r* = 0.34; *P* = 0.21; Spearman’s rank correlation) or July 2008 (*r* = − 0.07; *P* = 0.67; Spearman’s rank correlation). On the contrary, the ratio of brood cells to adult bee combs (NC/P) in July was significantly greater in the control CS and C groups than in the treated groups (ANOVA, F = 6.7, *P* < 0.001; Figure 4C). By contrast, in August only the CS group exhibited a significantly higher NC/P ratio with respect to groups 1 T (*P* = 0.004), 2 T (*P* = 0.03) and 4 T (*P* = 0.006).

##### Natural queen supersedure and colony mortality

Only 4 of the 50 colonies underwent natural queen supersedure during the study: 2 colonies in group 1 T (colonies 1 T-6 and 1 T-8), 1 from group 4 T (4 T-9) and 1 from the C group (C-7) in June 2008, while colony C-3 changed its queen in July 2008 (see Additional file 1).

Several colonies died before the end of the study in December 2008. One colony from group 4 T collapsed in February 2008 with signs of chalkbrood disease. Another colony from group 4 T and one of group 1 T died in July and May 2008 respectively after one month producing only drone brood (queenless or laying worker colony; see Additional file 1).

Three CS colonies died in the winter-spring of 2008, one with symptoms of American Foulbrood (AFB) prior to collapse, while no bees remained inside the colony after collapse in the other 2. In group C, 3 colonies died during the same period, 2 of which only produced drone brood before collapse. In these colonies no death or crawling bees were detected around the hives or faecal marks.

In the analyses prior to collapse, all the dead colonies contained a percentage of *N. ceranae* infected forager bees of over 45% (range 45-95%).

In the period following interventions in the colonies, 2 colonies from the 1 T group collapsed, exhibiting symptoms of AFB in January 2009. Of the untreated colonies, 4 CS and 3 C colonies died between July 2009 and October 2010, leaving no bees in the hive (see Additional file 1). Thus, in December 2010 the percentage of surviving colonies was 70% in group 1 T, 100% in group 2 T, 80% in group 4 T, 30% in group CS and 40% in group C.

##### Honey production

**Figure 5:**
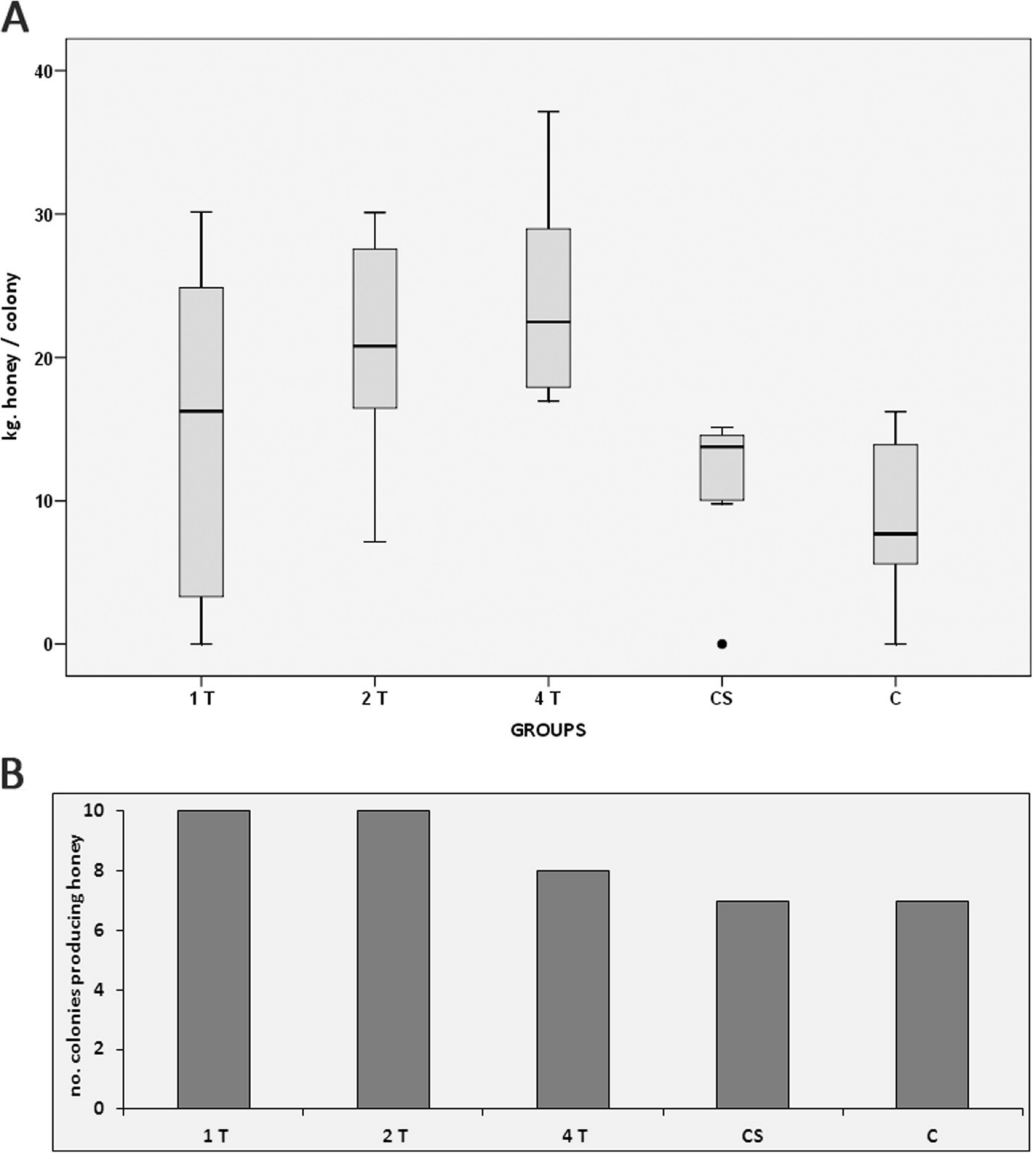
**Honey production in the colonies studied. A**: Average honey production (kg) per group upon harvesting (September 2008). **B**: Number of productive colonies per group in the harvesting season (September 2008).

The colonies of group 4 T produced the highest amount of honey (24.1 ± 7.3 kg honey/colony), with a similar level to that achieved by group 2 T colonies (21.2 ± 7.3 kg h/c; Figure 5A). Both groups were significantly more productive (*P* < 0.05) than groups CS (11.5 ± 5.1 kg h/c) and C (8.9 ± 5.9 kg h/c). The colonies in group 1 T produced less honey (14.5 ± 12.1 kg h/c) than the colonies in the other treated groups but more than the control ones, although these effects failed to reach statistical significance (*P* > 0.05).

The amount of honey produced by each colony in September 2008 was significantly related to the percentage of *N. ceranae* parasitized bees in the corresponding hives in July 2008 (*r* = − 0.51, *P* = 0.002; Spearman’s rank correlation), but not in November 2007 (*r* = − 0.29, *P* = 0.09; Spearman’s rank correlation). On the contrary, no correlation was found between the honey production and the percentage of *N. apis* parasitized bees in November 2007 (*r* = − 0.28, *P* = 0.11; Spearman’s rank correlation) or July 2008 (*r* = − 0.21, *P* = 0.14; Spearman’s rank correlation).

The colonies that died before the harvesting season (2 colonies from group 4 T, 3 colonies in group CS and 3 colonies from group C) were not considered when calculating the mean honey production of each group (Figure 5B).

## Discussion

In this field study, we confirm the negative influence of *Nosema* infection on adult honey bee population size and brood production, and we show that when these microsporidia are not controlled, infection provokes a significant decrease in honey production. Since *N. ceranae* was the dominant species in the infected colonies throughout the study, the observed effects may principally be attributed to this microsporidium. However, although *N. apis* shows a small prevalence within the infected colonies and was not constantly present in them throughout the assay, a negative effect of this microsporidium over the colonies health cannot be discarded.

The number of adult bees, sealed brood and the amount of honey produced are valuable indicators of hive health [38]. In our experimental conditions, the health status of the colonies was worse when they were highly infected with *N. ceranae* than when infection was controlled, suggesting that this microsporidium may have deleterious effects at the colony level, as suggested previously [17,39,40]. It is also worth mentioning that part of the non-treated colonies died leaving no bees in the hive at the moment of collapse, coinciding with signs described in a preceding study [17]. Therefore, the parameters here presented regarding hive health status, taken together with other recently shown [36,41,42], may be considered as sub-clinical signs of nosemosis type C. However, as the number of colonies per group and sample sizes were limited, definitive conclusions cannot be made and the results presented here should be supported by more research.

The percentage of *Nosema*-infected forager bees is thought to be a potentially useful indicator of the extent of colony infection [12,17,43,44]. The proportion of *Nosema-*infected forager bees in our colonies revealed significantly stronger infection than in the untreated colonies (groups CS and C) after the autumn and spring interventions, which may account for the observed differences in the fitness parameters like population size, brood production and honey production from those of the treated colonies (groups 1 T, 2 T and 4 T). Moreover, after the spring intervention we detected a significant negative correlation between *N. ceranae* infection and the size of the worker population, as seen previously [17,39]. Indeed, there were significantly fewer bee combs in the spring and summer months in the colonies with the strongest *N. ceranae* infection (groups CS and C). Colony population size depends mainly on the worker bee life span [11] and as *N. ceranae* has a negative impact on this parameter in honey bees [21,45,46], it probably exerts a direct negative impact on colony size in accordance with our results.

*Nosema* infection was less prevalent in house bees than in forager bees, as reported previously [17,35,47]. Since the probability of acquiring infection is likely to increase with age and novel diseases are more often picked up by foragers, young workers should be less likely to be infected [48]. A direct link has been reported between infection levels in forager and house bees [49,50], but in the present assay we failed to establish such a correlation as the number of colonies in which the proportion of infected house bees was analysed was very limited. However, a subsequent study suggested that the interior bees may more accurately reflect the infection level within the colony [36]. In our experimental conditions, forager bee infection rates of at least 45% were shown in all the collapsed colonies, while the percentage of infected house bees in collapsed colonies, only measured in colonies CS-7 and 4 T-6, was 20% and 35%, respectively. However, infection levels of 35% were also recorded in house bees in a colony that did not collapse (colony C-3) in July. This particular colony underwent natural queen supersedure in July, which may have subsequently decreased its infection rate and thus, the likelihood of collapse [36]. Yet as the infection rate was not monitored after this time point, this hypothesis could not be corroborated. In colony C-6, which also underwent queen supersedure in June, the infection rate in house bees after one month was lower than that observed in the treated colonies (15%), while the forager bees exhibited a very high level of *Nosema* infection (75%). Thus, the influence of queen replacement on *Nosema* infection appears to primarily affect the house bee population, and the foragers to a lesser extent, although again this hypothesis could not be confirmed since infection rates were not subsequently measured.

Brood production was not correlated with infection levels, although less infected colonies had significantly more brood cells than those in highly infected groups at different time points throughout the study, consistent with earlier studies of *Nosema-*infected colonies [17,29,51]. Furthermore, brood production was slower in the untreated groups (groups CS and C), peaking in July, than in groups 1 T, 2 T and 4 T, which began growing earlier and reached maximum levels one month earlier (June).

Adult bee population size and brood production are correlated [39,52,53], and as mentioned above, increased bee mortality has been described in *N. ceranae-*infected bees. The smaller populations observed in strongly infected colonies may therefore reflect a combination of decreased brood rearing and increased forager death in these colonies. Furthermore, thermoregulation and brood food production may be dysregulated in infected colonies with small populations [54], resulting in reduced brood rearing efficiency [55]. Nurse bees begin to forage precociously in response to high forager death rates [54,56-58]. While this strategy restores the proportion of foragers in the population, it also shortens the overall lifespan of adult bees [59-61], as well as their effectiveness and resilience as foragers [62]. This strategy also reduces the time each bee can dedicate to colony growth and brood production. Thus, when the rate of brood production is insufficient to replace the forager bees lost due to *Nosema* infection, the decline of the colony may accelerate [17,58].

Colonies with high levels of *N. ceranae* infection throughout the study (groups CS and C) had significantly more brood cells relative to the adult bee population during the summer months (July and August). As the bee colony grows, a decrease in the ratio of brood to bees has been reported previously [53,63,64], as observed during the evolution of the colonies with a milder degree of infection here (groups 1 T, 2 T and 4 T). By contrast, strongly infected colonies (groups CS and C) did not grow at the same rate as treated colonies, and they did not experience a similar decrease in the proportion of brood to bees. The higher ratio of brood to bees in severely infected colonies may reflect the increased rearing effort in the colony in an attempt to replace the worker bees lost due to *Nosema* infection [29], although further studies will be necessary to confirm this hypothesis.

Since honey production is generally correlated with the number of worker brood cells and the worker population [27,65], larger colonies tend to store more honey [66]. In addition, nectar foragers from larger colonies visit more flowers on each trip, with a shorter handling time per flower when compared to those from small colonies [67]. However, *Nosema* may negatively influence the flight ability of infected honey bee foragers [68,69] and as such, the value of the nectar and pollen resources will depend on the status of the colony [70]. According to our results, colonies with more severe *N. ceranae* infection, with smaller colonies and brood area, had lower yields of honey, as reported previously for *N. apis-*infected colonies [12,51].

Although there was no correlation between *N. ceranae* infection and variations in colony strength or increased winter mortality in several recent studies [26,71,72], in our experimental conditions this microsporidium was very pathogenic to honey bee colonies, as shown by its impact on population size, the amount of brood and honey production. These conflicting findings may reflect differences in the experimental procedures used, as different methods and time points were used when measuring infection, colony strength and other parameters related to colony health. Accordingly, it may be necessary to standardise the procedures to evaluate *Nosema* infection in honey bee colonies in order to accurately compare the results from different studies and regions, and to better understand the differences in the epidemiology and pathology of this microsporidium worldwide.

The treatment used against *Nosema* in the present study (Fumidil B^®^) showed to be successful at temporarily reducing *N. ceranae* infection levels in the colonies, as demonstrated previously [17,73], and allowed us to compare the health status of strongly and mildly infected colonies.

Collapsed colonies (2 in group 4 T, 3 in group CS and 3 in group C) exhibited severe *N. ceranae* infection prior to collapse, consistent with previous reports [17,74]. The collapse of treated colonies in group 4 T may have occurred due to poor fumagillin consumption and hence, limited efficacy [33]. Alternatively, in-hive conditions in this group may have been severely disturbed due to the administration of fumagillin in the winter time (unlike groups 1 T and 2 T), triggering a deadly infection by this microsporidium. Otherwise, since this group received the treatment more frequently, a negative impact of this molecule over colony health may have occurred [40], but all these suggestions should be studied more. One of the collapsed colonies of group 4 T was affected by chalkbrood disease, while another colony from the CS group that died exhibited symptoms of foulbrood disease. *N. ceranae* infection in these colonies may have provoked the outbreaks of stress-related diseases such as chalkbrood [42] and foulbrood disease. However, the surviving colonies in the CS and C control groups presented high levels of *N. ceranae* infection throughout the study, remaining alive but undergoing the effects of a chronic disease, as demonstrated by the reduced levels of fitness components such as colony size, brood rearing and honey production.

Despite the remarkable impact of *Nosema* infection on beekeeping economics [11,12,29], this effect is often underestimated by beekeepers [75]. However, in our experimental conditions *N. ceranae* infection was highly pathogenic to honey bee colonies, as witnessed by sub-clinical signs such as significant decreases in colony size, brood rearing capacity, honey production and, as reported previously [17,74], a decrease in the rate of survival. The study revealed that the control of *Nosema* in autumn and spring could be enough to mitigate these negative effects of nosemosis type C on colony health and productivity, which in turn may affect beekeeping profitability and have dramatic consequences on crop pollination and in natural ecosystems. As treatments for *Nosema* are currently unavailable in many countries, further studies of potential treatments or beekeeping techniques are urgently required to combat the rapid spread of this dangerous emerging disease.

## Competing interests

The authors declare that they have no competing interests.

## Authors’ contributions

MH, RMH, AM conceived and designed the experiments. CB, MH, RMH performed the experiments and analysed the data. LB, CB, MH performed the statistical analysis. CB, MH, RMH wrote the paper. All authors read and approved the final manuscript.

## Supplementary Material

Additional file 1 **Natural dynamics of *Nosema *sp. infection in the colonies of the assay and other remarks.** This figure shows PCR amplification of forager bee composite samples (*n* ≥ 30 bees per sample) throughout the study and the data describing other pathologies detected, natural queen supersedure events and colony collapse. Click here for file

Additional file 2 **Map of the area where the experimental apiaries were located (Source: screenshot of Google™ Earth).** The two experimental apiaries were situated 500 m away from one another and were surrounded by the same type of flora. Click here for file

Additional file 3 **Percentage of parasitised forager bees per group before and after the interventions in autumn 2007 and spring 2008.** Significant differences between groups were determined for each period. Footnotes: Significant difference with respect to CS: (*P* = 0.007)^a^; (*P* ≤ 0.006)^b^; (*P* ≤ 0.001)^c^; (*P* ≤ 0.001)^e^; (*P* ≤ 0.001)^g^; (*P* = 0.04)^i^; (*P* = 0.001)^m^; (*P* ≤ 0.001)^o^; (*P* ≤ 0.001)^q^. Significant difference with respect to C: (*P* ≤ 0.001)^d^; (*P* ≤ 0.001)^f^; (*P* ≤ 0.001)^h^; (*P* = 0.02)^j^; (*P* = 0.01)^n^; (*P* ≤ 0.001)^p^; (*P* ≤ 0.001)^r^. Significant difference with respect to 1 T: (*P* ≤ 0.001)^k^; (*P* ≤ 0.001)^l^. Click here for file

Additional file 4 **Mean proportion of infected forager bees (*n* = 20 bees per colony) before and after interventions in autumn 2007 for each group.** Asterisk indicates significant differences (*P* < 0.001) in the pre-post intervention interval. Click here for file

Additional file 5 **Mean proportion of infected forager bees (*n* = 20 bees) before and after interventions in spring 2008 in each group.** No significant differences (*P* > 0.05) were detected in any group in the pre-post intervention interval. Click here for file

Additional file 6 **Number of bee combs (± s.d.) and number of brood cells (± s.d.) throughout the assay.** This table shows the average number of bee combs and average number of brood cells per group in each time point of the assay. Click here for file
