# Supplementary figures and images for: Nosema spp. infection and its negative effects on honey bees (Apis mellifera iberiensis) at the colony level
Source: Vet Res. 2013 Apr 10;44(1):25. doi: 10.1186/1297-9716-44-25 (PMC3640932; doi:10.1186/1297-9716-44-25)

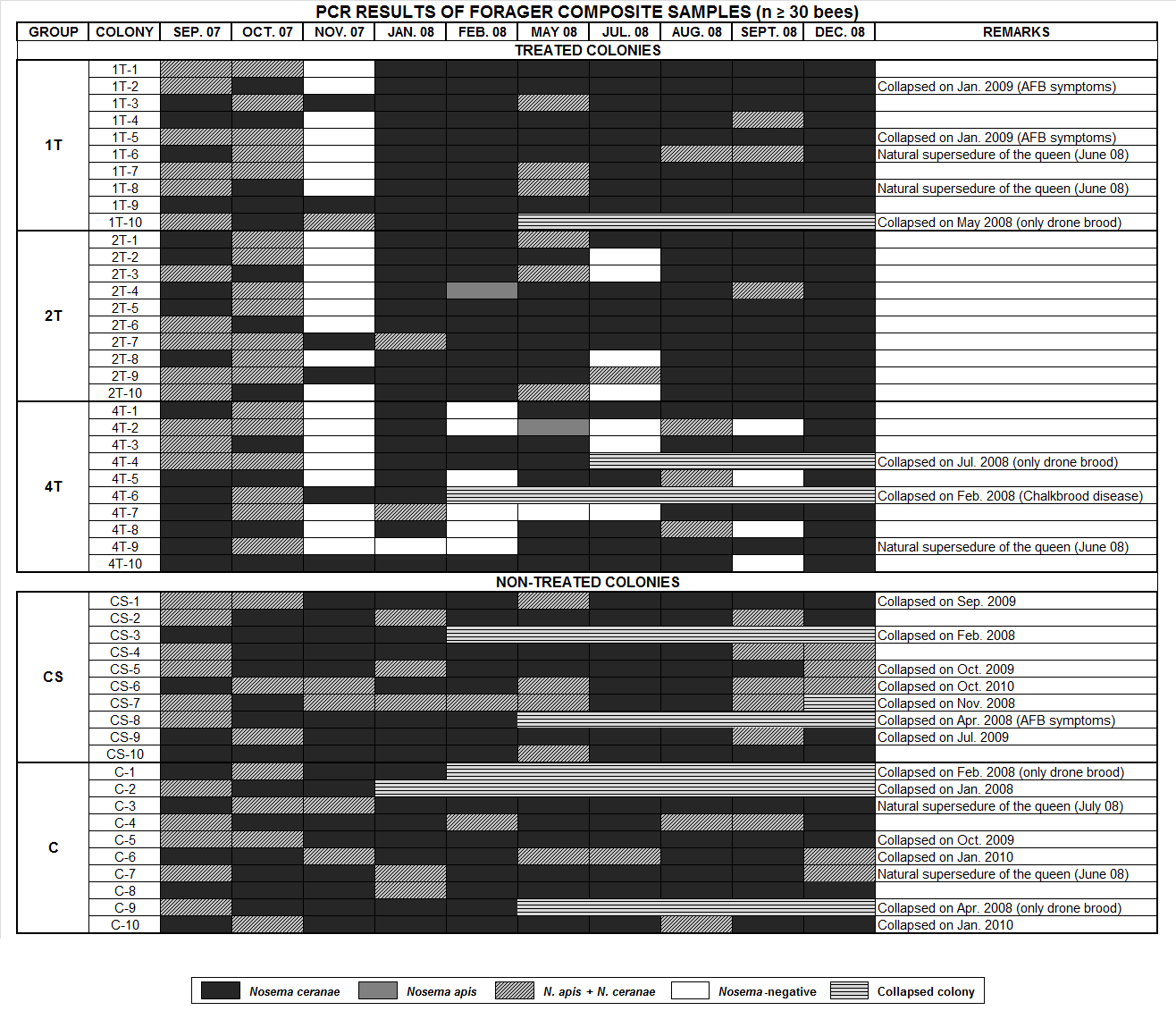

Supplement: Additional file 1 — Natural dynamics of Nosema sp. infection in the colonies of the assay and other remarks. This figure shows PCR amplification of forager bee composite samples (n ≥ 30 bees per sample) throughout the study and the data describing other pathologies detected, natural queen supersedure events and colony collapse. [file 1297-9716-44-25-S1.tiff]

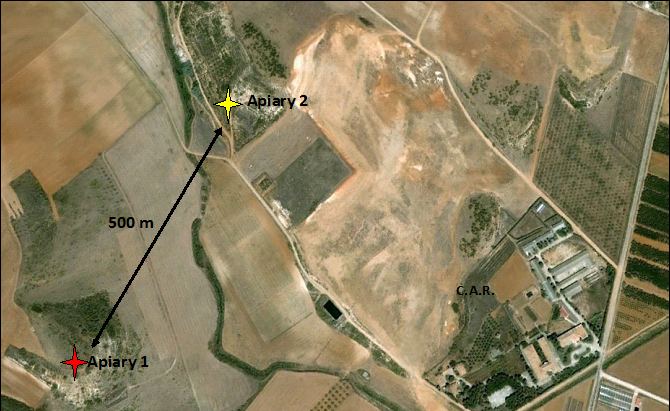

Supplement: Additional file 2 — Map of the area where the experimental apiaries were located (Source: screenshot of Google™ Earth). The two experimental apiaries were situated 500 m away from one another and were surrounded by the same type of flora. [file 1297-9716-44-25-S2.tiff]

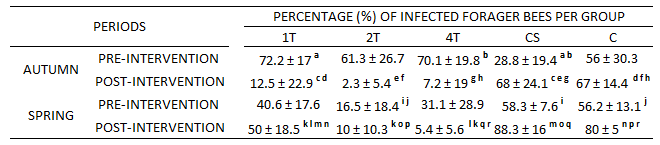

Supplement: Additional file 3 — Percentage of parasitised forager bees per group before and after the interventions in autumn 2007 and spring 2008. Significant differences between groups were determined for each period. Footnotes: Significant difference with respect to CS: (P = 0.007)a; (P ≤ 0.006)b; (P ≤ 0.001)c; (P ≤ 0.001)e; (P ≤ 0.001)g; (P = 0.04)i; (P = 0.001)m; (P ≤ 0.001)o; (P ≤ 0.001)q. Significant difference with respect to C: (P ≤ 0.001)d; (P ≤ 0.001)f; (P ≤ 0.001)h; (P = 0.02)j; (P = 0.01)n; (P ≤ 0.001)p; (P ≤ 0.001)r. Significant difference with respect to 1 T: (P ≤ 0.001)k; (P ≤ 0.001)l. [file 1297-9716-44-25-S3.tiff]

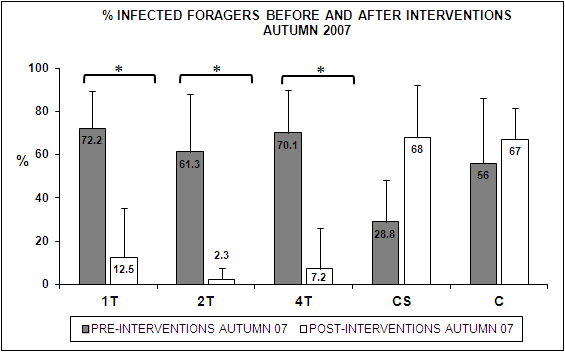

Supplement: Additional file 4 — Mean proportion of infected forager bees (n = 20 bees per colony) before and after interventions in autumn 2007 for each group. Asterisk indicates significant differences (P < 0.001) in the pre-post intervention interval. [file 1297-9716-44-25-S4.tiff]

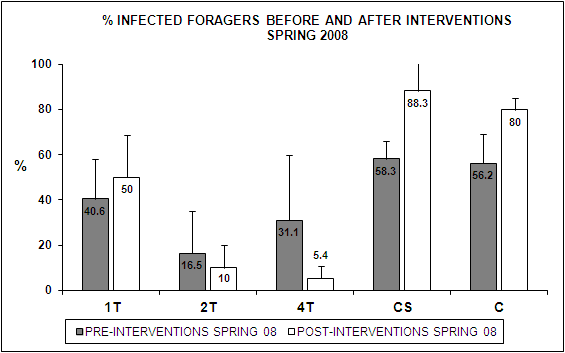

Supplement: Additional file 5 — Mean proportion of infected forager bees (n = 20 bees) before and after interventions in spring 2008 in each group. No significant differences (P > 0.05) were detected in any group in the pre-post intervention interval. [file 1297-9716-44-25-S5.tiff]

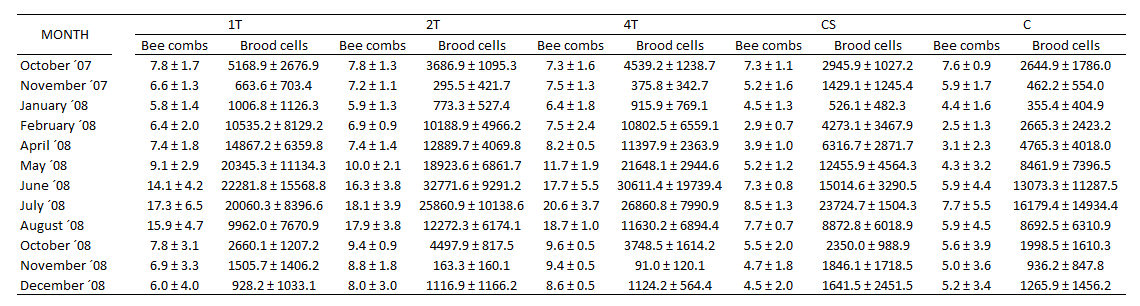

Supplement: Additional file 6 — Number of bee combs (± s.d.) and number of brood cells (± s.d.) throughout the assay. This table shows the average number of bee combs and average number of brood cells per group in each time point of the assay. [file 1297-9716-44-25-S6.tiff]
